# Testing hypotheses about the harm that capitalism causes to the mind and brain: a theoretical framework for neuroscience research

**DOI:** 10.3389/fsoc.2023.1030115

**Published:** 2023-06-19

**Authors:** Danae S. Kokorikou, Ioannis Sarigiannidis, Vincenzo G. Fiore, Beth Parkin, Alexandra Hopkins, Wael El-Deredy, Laura Dilley, Michael Moutoussis

**Affiliations:** ^1^Psychoanalysis Unit, Department of Clinical, Educational and Health Psychology, University College London, London, United Kingdom; ^2^Institute of Cognitive Neuroscience, University College London, London, United Kingdom; ^3^Department of Psychiatry, Icahn School of Medicine at Mount Sinai, New York, NY, United States; ^4^Department of Psychology, School of Social Sciences, University of Westminster, London, United Kingdom; ^5^Department of Psychology, Royal Holloway, University of London, London, United Kingdom; ^6^Centro de Investigación y Desarrollo en Ingeniería en Salud, Universidad de Valparaíso, Valparaíso, Chile; ^7^Department of Communicative Sciences and Disorders, Michigan State University, East Lansing, MI, United States; ^8^Wellcome Centre for Human Neuroimaging, University College London, London, United Kingdom; ^9^Max Planck University College London Centre for Computational Psychiatry and Ageing Research, London, United Kingdom

**Keywords:** capitalism, inequality, neurodiversity, deprivation, isolation

## Abstract

In this paper, we will attempt to outline the key ideas of a theoretical framework for neuroscience research that reflects critically on the neoliberal capitalist context. We argue that neuroscience can and should illuminate the effects of neoliberal capitalism on the brains and minds of the population living under such socioeconomic systems. Firstly, we review the available empirical research indicating that the socio-economic environment is harmful to minds and brains. We, then, describe the effects of the capitalist context on neuroscience itself by presenting how it has been influenced historically. In order to set out a theoretical framework that can generate neuroscientific hypotheses with regards to the effects of the capitalist context on brains and minds, we suggest a categorization of the effects, namely deprivation, isolation and intersectional effects. We also argue in favor of a neurodiversity perspective [as opposed to the dominant model of conceptualizing neural (mal-)functioning] and for a perspective that takes into account brain plasticity and potential for change and adaptation. Lastly, we discuss the specific needs for future research as well as a frame for post-capitalist research.

## Introduction

In this paper, we outline the key ideas behind a theoretical framework for neuroscience research that reflects critically on the neoliberal capitalist context. We argue that neuroscience can and should illuminate the effects of neoliberal capitalism on the brains and minds of the population living under such socioeconomic systems.

To illustrate, millions of US citizens have very poor access to mental health provision (Agency for Healthcare Research Quality, 2015). In the UK, Mental health suffered greatly under the austerity policies from 2008 onwards, with people living in poverty being affected the most (Cummins, 2018). Another example is offered by Greece, whose debt crisis and failure as a capitalist economy, jointly with the policies required by the IMF, the European Central Bank and the European Union (IMF, ECB and EU) for the “rescue” of its market, led to a mental health crisis (Karanikolos et al., 2013; Parmar et al., 2016).

Such examples are well-documented and widely known, but to bridge the gap with neuroscience research, there is a need for a cohesive theoretical framework that assumes a critical perspective on key features of current politics and ethics (see Box 1). We posit that neuroscience research is dominated by the discourses of free-market agents; of medicalization of suffering; and of pathologizing of individuals who are unable to live up to the free market standards of independence. This results in the reproduction of harmful practices in mental health, and the dissemination and popularizing of harmful misconceptions about health and illness. We posit that the concept of neurodiversity is both more inclusive, since it allows neurodivergence to be examined but not pathologized (Kirmayer et al., 2015; Craine, 2020; Kapp, 2020), and scientifically more promising, in view of the substantial empirical evidence that the validity of many psychiatric diagnostic categories is poor (Insel et al., 2010; Cuthbert and Insel, 2013; Peng et al., 2021).

Box 1 **Methodological note.**

The methodology through which this article was developed rested first, on a brief scoping review of the literature, based on forward and backward searchers centered on key articles. We found that there was a huge wealth of epidemiological findings linking modern conditions with mental health, especially poverty, sometimes appealing to neurobiological findings, but in a very indirect, narrative way rather than specific psychobiological hypotheses. Second, we took into account the critique that the human sciences have to offer to the practice of neuroscience and biomedicine, and noticed that these too shied away from moving from a deductive-theoretical to a falsificationist framework, for fear of positivist contamination. Hence, we used our own expertise and targeted, iterative literature searchers to address these points not in terms of assertion, but of research methodology. Third, we acknowledge that the meaning of the concepts we study here is often controversial. Rather than claiming that our own definitions are good enough, we detail the sense in which we use the terms in question in the Supplementary material. Finally, we made use of clinical experience and inspiration from discussions with experts-by-experience of symptoms. The latter belonged to two Lived Experience Advisory Panels convened to shape neuroscience research questions (incl. Humm et al., 2020). They also included discussions with neurodiverse scientists in the context of a relevant workshop on neurodiversity and research in the first Princeton Conference on Computational Psychotherapy, 2022. We are very grateful to these experts for inspiring discussions.

Further to this, we highlight neuroplasticity as the other side of this coin, disproving the common assumption that neuroscience must be the scientific arm of the biomedical model to mental health. We also emphasize the peril of invoking the concept of neuroplasticity in the presence of the intense flexibility and adjustability that is required in the context of the globalized, neoliberal economy. Finally, we discuss requirements for an emancipatory neuroscience, we make specific suggestions for further research, and suggest a framework for post-capitalist, society and community-oriented research.

## Harmful influences to mind and brain in capitalism

### Harm to mind

One of the key detrimental influences of capitalism on mental health is the production of inequality. For example, in the 30 years following 1977, 60% of the increase in US national income went to just the top 1% of earners; this is projected to become even more extreme without tax adjustment (Piketty, 2015). Piketty argues that inequality is not an accident but an intrinsic feature of capitalism (Piketty, 2013), while free-market economists argue that substantial inequality is good for the economy (Hasanov and Izraeli, 2011) and advocates of capitalism promote inequality as a great good (Tamny, 2016). Thus, the prevailing view is that capitalist policy-making creates inequality.

The link between inequality and mental health is well-documented (Mangalore et al., 2007; Rao et al., 2012; Patel, 2020). However, the precise mechanisms mediating the interaction between gender, identity, economic inequality and poor mental health are uncertain. It has been suggested that associations between poverty and mental health are not just a matter of material living conditions, but mediated by relative poverty or inequality, this being fundamentally a “relation between people” (Wilkinson and Pickett, 2017, 2018).

Layte (2012) explored various hypotheses about how income inequality may result in poorer mental health which provided some evidence about the role of social rank and anxiety as well as social capital and the resulting interpersonal and institutional trust and capacity to cooperate.

Many researchers remark that there is an alarming lack of in-depth and methodologically sound research into the complex effects of capitalism on the least well-off (Burns, 2015; Piketty, 2015). The effects of poverty are often studied in the context of low socioeconomic status and neighbor effects (Sharkey and Faber, 2014). Such studies have provided evidence that socioeconomic disadvantage is associated with higher prevalence of psychological distress, measured as psychiatric disorders (Richardson et al., 2015). One possible pathway is that higher rates of deprivation (Table 1) expose individuals to multiple stressors, resulting in high prevalence of depression and anxiety (Ross, 2000; Galea et al., 2007). Such stressors include local crime (Astell-Burt et al., 2015), childhood trauma (Heim et al., 2008; Lupien et al., 2009) and job insecurity (Meltzer et al., 2010). At the same time, school status may explain variation in depressive symptoms better than neighborhood deprivation (O'Campo et al., 2015), in line with the idea that the effect of one's neighborhood process may be more or less relevant to an individual depending on the stage of their life (Ellen and Turner, 1997) and the specific network of relationships within their community.

**Table 1:** Deprivation, isolation, and intersectional effects of capitalism.

**Effect**	**Socioeconomic process**
**Deprivation effects**	Generative process central to capitalism
Material	Resulting from the marketization of basic needs or rights into profitable commodities, such as access to clean water, shelter, education, and healthcare. Lacking access to such needs can directly impact mental health but can also have an indirect impact via increased stress and reduce the overall quality of life.
Psychological	Deprivation over basic psychological needs will also exert detrimental effects on mental health. Include a lack of autonomy over one's time (e.g., via reduction of salaries and overworking), lack of job security and exposure to demeaning working conditions (e.g., deprivation of human dignity).
**Isolation effects**	Generative process central to capitalism
Workplace relations	Resulting from the enforcement of individual competition, instead of cooperation, as the means for ordinary individuals to survive and advance. This pervades how work relations are structured, as well as the general relations within the society, with detrimental effects for the mental health.
Community relations	These include mental health effects arising from the destruction of communities, of physical, virtual and symbolic communal space and the degradation of collective groups (e.g., Unions). This is also the resulting from the physical displacement of the workforce (i.e., internal and international migration) and insecure working conditions (e.g., gig economy).
**Intersectional effects**	Oppressive structures interacting with the capitalist context
Discrimination based on beliefs, ethnicity, gender, sexuality, etc.	These stigmatizing practices are worsened under capitalism, which relies on discriminations to exploit segments of the workforce and reduce production costs. These strategies have been linked with chronic high-threat levels, as well as increased deprivation and isolation effects, with harmful consequences to mental health.
Discrimination due to financial dependence	Being stigmatized as unsuccessful in an unrealistic representation of individual responsibilities and agency in pursuing financial success, which results in individuals being denied access to essential services (e.g., housing, credit etc.). This results in further worsening deprivation and isolation effects, with negative consequences for mental health.

Another factor may be that neighborhood disadvantage is associated with fewer resources to help individuals with mental health needs and weaker social support networks, exacerbating depressive and anxious symptomatology (Cutrona et al., 2006; Gariepy et al., 2015). Additionally, increased exposure of residents from disadvantaged neighborhoods to substance abuse has been linked to increased substance abuse disorders and associated mental health problems (Galea et al., 2004). Consistent with this perspective, some evidence indicates that moving away from highly deprived areas is associated with mental health benefits (Leventhal and Brooks-Gunn, 2003; Osypuk et al., 2012), supporting a causal relationship between low socioeconomic status and mental distress.

Ultimately, part of these detrimental effects could be offset by investing significant resources in welfare state, to provide free, universal access to essential services such as housing, clean water, education and healthcare, thus significantly reducing risk of physical and mental distress. However, such regulatory interventions conflict radically with the neoliberal ideology, where the free market is assumed to be necessary for the optimal allocation of goods and services. This ideology, which is not supported by historical data, has pushed toward the commodification of basic needs, to the detriment of the quality of life and the mental health of large swaths of the population. By not alleviating and by allowing the increasing inequalities to perpetuate, capitalism is perpetuating the deleterious effect of poverty on the mind and brain (Muntaner et al., 2007).

Stigmatization is also crucial. Although far from originating in or being specific to capitalism, it is likely to be an important process used by capitalism to enhance inequalities (Table 1). Stigmatization is negative labeling and discrimination through exertion of social power (Hatzenbuehler et al., 2013). Thus, any socio-economic system within which difference (or diversity) is linked with power is likely to stigmatize certain groups. Current neoliberal discourses are selective as opposed to intersectional i.e., they may condemn stigmatization along some axes of identity (e.g., condemning sexism), but they continue to stigmatize identities associated with impaired productivity and class. Thus, those with disabilities and mental health problems are particularly prone to being labeled as free-riders and other psychologically harmful categories in market societies (Scambler, 2018). Stigma can result in reduction of purely economic opportunity, but critically it results in psychological harm (Zeng et al., 2018). A specific form of stigma found in capitalism is attached to those that “fail” in the market, be it simply through poverty (Yang and Walker, 2020) or through failed entrepreneurship (Engel and Pedersen, 2019).

The relationship between socioeconomic status and mental health does not seem to be exclusively linear. Individuals in the “middle” of the capitalist hierarchy [i.e., in so-called contradictory class locations (Wright, 1998)] may also experience high levels of distress. The term contradictory class location refers to the absence of adequate control over one's work that the top of the hierarchy enjoys, together with the experience of threat from lower strata (and peers). It has been found that individuals in the middle of the organizational hierarchy (such as managers, supervisors, salaried professionals and other contradictory class locations) tend to suffer higher rates of anxiety (Prins et al., 2015). This is important, as it highlights that class can affect one's mental health irrespective of economic status. It has been argued (Muntaner et al., 2007) that it may be social stratification based on characteristics other than social class that determines mental health outcomes, and studying the level of exploitation or a number of other class determinants may enrich this research area.

Academia may be an example of interest to the reader. PhD students and early career researchers (i.e., those in the middle of the organizational hierarchy) seem to be disproportionately affected by anxiety (Nature Editors, 2019), despite their prestigious position in society in general. Contributing factors may include academic capitalism (Jessop, 2018), the winner-takes-all ethos of academia alongside the exploitative culture of overwork, and the fact that the short doctoral and postdoctoral contracts allow many employers and supervisors to look the other way when it comes to a duty of care (Nature Editors, 2019).

Next, it is important to mention the growing role played by addiction in modern-day capitalist societies which also illustrates the inextricable link between brain and mind. Addiction is commonly defined as the maladaptive repetition of a certain behavior, despite adverse consequences (Kuznetsova, 2002; Redish et al., 2008; Dayan, 2009). This definition has been traditionally applied to the use of “substances of abuse” (e.g., nicotine, alcohol, illicit and prescription drugs, etc.), and more recently it has been extended to several other behaviors that are deprived of a direct pharmacological interference e.g., gambling, internet gaming, social media use etc. We have already mentioned the fact that capitalist societies are characterized by built-in inequality conducive of dysphoria, stress, and anxiety, which have long been known to foster addiction disorders (Volkow and Morales, 2015). In comparison with these forms of addiction, which can be considered an undesired by-product of the capitalist socio-economic structure, new forms have emerged that are actively pursued to generate the repetition of profitable behavior. The most evident examples of these strategies are offered by the development of new chemical compounds that artificially increase the addictive power of a known substance [cf. the case of nicotine (Rabinoff et al., 2007; Biswas et al., 2016)], or the use of incentive campaigns to promote the unnecessary prescription of highly addictive and dangerous drugs [cf. the case of opioids (Van Zee, 2009; Gray et al., 2021)]. Furthermore, the understanding that addictions are driven by dopaminergic responses to unexpected events (Redish et al., 2008; Smith et al., 2021) is being used to design schedules of reinforcements that increase the chances of repeated use, as in social platforms or online gaming (Lindström et al., 2021). This micro-level reinforcing of unhealthy behaviors for profit at the expense of health adds to the powerful lobbying and advertisement tools through which substances recruiting brain reward systems, such as sugar or nicotine, have been promoted (Freudenberg, 2014).

In the interest of space, we will only briefly mention here the (evident and evidenced, though not directly causal) effects of the fashion and media industry on women's identity and the links that have been made to eating disorders and body dysphoria (Levine and Murnen, 2009).

### Harm to brain

Epidemiological data regarding inequality and competition is suggestive, but the hypothesis that these forces cause neurobiological impairments requires accumulation of evidence regarding putative causal pathways, complemented by methodological debate regarding the limitations of each neuroscientific paradigm. Evidence from animal studies of stressors analogous to those found in capitalist labor markets demonstrate putative mechanisms, but generalization to human beings requires much additional research, for which we call for here. Cross-sectional human neuroscientific studies also provide evidence, albeit correlational, while longitudinal ones allow temporal separation of exposure (e.g., childhood disadvantage) and impairment (e.g., adult brain structure). Capitalist systems also provide “natural experiments”, especially boom-and-bust market dynamics, which afford within- as well as across- subject exposures, both for individuals and whole populations.

Free markets are stressful by construction, with some highly prevalent types of market stress having close parallels with animal laboratory stressors causing neurobiological harm. These include *unpredictable chronic mild stress* e.g., being in a demanding job with little control leading to psychiatric morbidity (Stansfeld et al., 1999), *social defeat* e.g., social defeat hypothesis of schizophrenia (Selten and Cantor-Graae, 2005; Selten et al., 2016), and *resource uncertainty* e.g., millennial precarity, academic precarity (Berg et al., 2016; Brunila and Valero, 2018; Worth, 2019). “Unpredictable Chronic Mild Stress” reliably causes anhedonia in rodents and is also known to affect brain structure and neurochemistry (Khan et al., 2018). Social defeat, whose human analog is endemic in capitalism, is well-known to cause brain impairments. In animal research, social defeat (SD) is the forced exposure of a less endowed index animal, usually a smaller male, to a dominant animal which proceeds to inflict species-specific (usually inter-male) aggression onto the index animal. SD impairs the brain acutely, e.g., reducing neurotrophin expression in rodents (Pizarro et al., 2004) while in its chronic form it impairs dopamine reward signaling (Cao et al., 2010). Resource uncertainty strikingly resembles the *variable foraging demand* paradigm, a form of stress that impairs primate neural development (Coplan et al., 2006). Here, mothers with infants are subjected to variability in nutritional resources, while these never drop to a mean level that could cause physical malnourishment. In capitalism, induction of insecurity is a key way in which work flexibility has been implemented. The mental and physical cost of this stress is contained by, or better externalized to, families and carers, so that feminized labor is exploited to palliate the harm caused by work insecurity (Watson, 2016). This hypothesis, that market subjects may suffer neurobiological wear analogous to laboratory subjects, should strongly motivate researchers to fill the very considerable gaps between animal and human neuroscience in this domain.

Pioneering work has been performed by Farah et al., who found that socioeconomic deprivation in childhood is associated with impaired function (e.g., executive function deficits in childhood), but also structural brain changes. Studies commonly find reductions in cortical and subcortical gray matter in children, some detectable as early as 5 weeks of age (Betancourt et al., 2016). Numerous studies have shown brain structure to depend on socioeconomic status, but the distribution of findings varies, possibly due to methodological reasons that plague brain imaging reliability in general (Farah, 2017). Nevertheless, large studies provide evidence that brain surface area is reduced in the presence of poverty (Noble et al., 2015).

**Figure 1:**
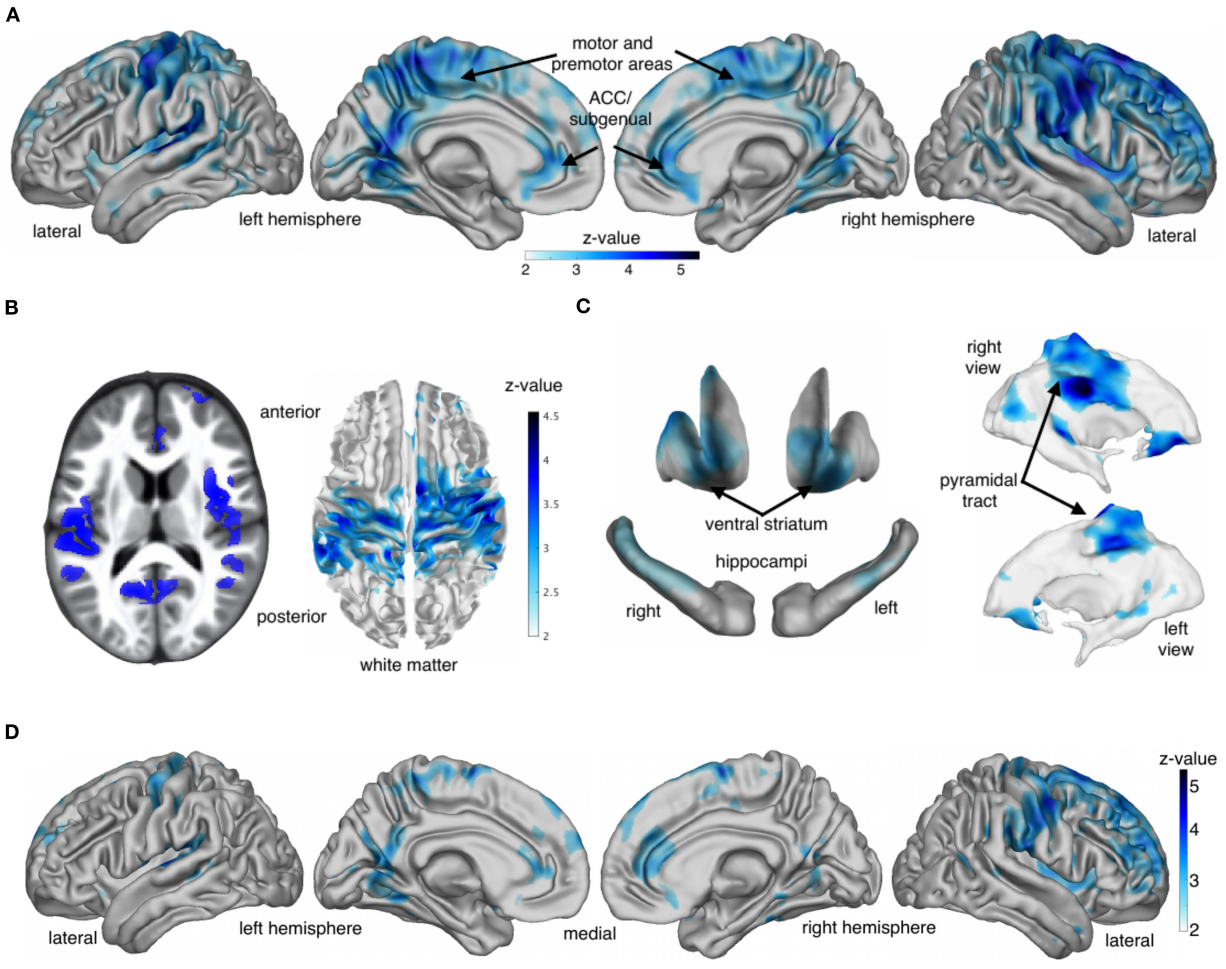
Based on Ziegler et al. (2020). In blue are the areas where socio-economic disadvantage before the age of 12, measured by an index of neighborhood poverty, is associated with a marker of slower growth of macromolecular content found in myelin, in a adolescent sample selected for low psychopathology. **(A–C)** Brain regions affected, without controlling for parental education. **(D)** Controlling for parental education, greatly reduced the effect, especially in medial cortical areas. Unexpectedly, controlling for parental occupation, a proxy for family income, made no impact, whether parental education was accounted for or not. **(A)** Reduce adolescent growth of cortical myelin-sensitive MT (over visits) with higher past disadvantage. **(B)** Also for MT within insula/operculum and cortex-adjacent white matter. **(C)** Subcortical regions and white matter core areas. **(D)** Reduced growth of cortical MT with early life SED controlling for parental education.

Another important, recent development is the introduction of neuroimaging sensitive to molecular processes, rather than just macroscopic anatomy. One study showed that growing up in a deprived neighborhood before the age of 12 was associated with slower growth of a myelin-sensitive marker in adolescence (Ziegler et al., 2020); that this was independent of a number of covariates such as IQ, parental occupation and positivity of parenting; but that it was partly mediated by parental education (see Figure 1). The sample in this study was screened for absence of psychiatric and neurological disorder, and no participants fulfilled the World Bank definition of poverty (not having enough to fulfill basic needs). This suggests that inequality within a neoliberal economy significantly impacts the brain structure of the children of those deprived of privilege (Kim et al., 2013; Dufford et al., 2020), although causality is to be demonstrated.

Research also connects brain-related measures affected by poverty and inequality to measures of mental function. The concept of IQ admits critique, but there is much evidence that it constitutes a reliable measurement of performance with clear brain correlates (Moutoussis et al., 2021). In turn, the development of IQ is impaired by poverty (Turkheimer et al., 2003; Hamadani et al., 2014), suggesting an adverse effect on the brain. Studies have started to demonstrate how the correlation between deprivation and IQ performance is mediated by specific brain features in advanced capitalist countries. McDermott et al. (2019) found evidence that reduction of the surface area of specific regions, such as inferior parietal and lateral temporal areas, mediates the relationship.

Not only brain structure, but also brain functional connectivity (FC) appears affected by economic disadvantage, the evidence mostly concerning young people from neoliberal societies. Here we should note that functional connectivity is a measure of “what the brain does”, specifically, what brain regions are organized into different networks, how active and how coherent these networks are. FC reflects both the biological structure of the brain but also the particular (mental) states occupied during scanning. Thus, despite being an entirely neurobiological measurement, it should not be seen as “neurobiological destiny”. In an important study, a neighborhood level measure of deprivation, the Area Deprivation Index, was associated with reduced FC in several brain networks (such as the Default Mode Network the Ventral Attention Network and others) in 9–11-year-old US children. In turn, the areas of reduced connectivity were associated with general cognitive, internalizing and externalizing symptom scores (Rakesh et al., 2021). Another study found that parental education was associated with a general decision-making ability, which was in turn associated with reduced functional connectivity in opercular, posterior cingulate and other FC networks (Moutoussis et al., 2021). This study, which controlled for participant IQ abilities, did not find an association with neighborhood-level deprivation.

### Toward a critical theoretical framework

Capitalism, currently in its neoliberal, globalized stage, is inextricably linked with inequality, but more importantly for us, it constitutes the context in which brains exist, develop, and function. Approaching the study of the brain from this angle allows us to use the rich theoretical work established by epidemiology, sociology, political science, critical theory, feminist and queer studies and many more, in order to formulate specific, empirically testable hypotheses.

Capitalist societies inculcate and enforce the idea that working relations must be based on competition to maximize profit and to promote meritocratic incentives. The boundaries of the resulting working relations are ill-defined (especially in unregulated neoliberal markets) and hence incentivize exploitation. Profit motivates capitalists to find ways to pressurize or tempt (as yet legally) unprotected individuals, in order to gain competitive advantages, leading to *deprivation effects* (Table 1). Furthermore, it is central to capitalism that enterprise ever expands into new areas, constantly creating groups deprived in relative or absolute terms, whose mental health it endangers. For example, when intercontinental slavery became possible, slave markets boomed. When (and where) child labor becomes profitable, child labor is marketed. When women and workers in caring roles are vulnerable, reproductive labor, that is, work maintaining the human potential of society, is unfairly exploited (Adams, 2020).

The capitalist appeal to a supposed meritocracy disempowers those “losing” within the system, as a dominant narrative dictates “if you try hard enough you will succeed”, promoted despite (and to counter) evidence that inequality is an enduring characteristic of the system (Piketty, 2013, 2015), thus assigning personal responsibilities for unavoidable features of the current social structures. Here the ideology sanctifying competition meets with its harmful result in lived experience, contributing to isolation effects (Table 1).

### The effects of the capitalist context on neuroscience

We now turn to the effects of the capitalist context on the basic, if unspoken, research assumptions and hypotheses that neuroscience works with.

Ever since the brain was suggested to be the seat of thoughts, emotions and behaviors, cultures have used their own paradigmatic metaphors to describe its functioning, appealing to the values, scientific and technological discourse of their time. From the automatons of the 17th century to the time of the train network and the electric circuit that lead to the Hodgkin and Huxley Nobel prize (Hodgkin and Huxley, 1952), and from the computer metaphor to machine learning, conceptualizations of the brain have always been heavily affected by and constructed within the socio-cultural context of their time. Therefore, it is not surprising that the capitalist way of thinking has permeated the modern fields of neuroscience.

Capitalism is defined as a socio-political system in which trade and industry are controlled by private owners for profit (Simpson and Weiner, 1989). More relevant to our times, Neoliberalism refers to the policies of economic laissez-faire and market deregulation. These include privatization, globalized free trade, and in general the compression of the role played by any collective or social entity (e.g., Governments, Unions, etc.), either as resource allocator or as a regulator of common interests. This favors supposedly optimal^1^ self-organizing, private actors, be they workers, multinational corporations or anything in between. The neoliberal economic paradigm, jointly with the theoretical framework that justifies its policies since the 1970s (Springer et al., 2016), affects almost every aspect of 21^st^ century life, with important ramifications for the psychology of people living within its parameters.

For the past few decades, healthy brains were often considered to be individualist and selfish computational machines, which process information in order to maximize personal utility via decision-making. That is, when faced with a decision, the decision-maker will make a cost-gain analysis and will decide based on self-interest (Osborne, 2004). This perspective both fits with and feeds into the postulated mechanisms controlling the interactions among agents and environment, as assumed in neoclassical economics and Rational Choice Theory as it was described by the “Chicago School of Economics” (Becker, 1976; Weintraub, 1993). In several countries, this view dominated, and turned theories of social, cognitive, behavioral, and neuroscientific research seeking to describe phenomena into prescriptions of how people should attain well-being through selfishly rational decision-making. More recently these decisions are assumed to be characterized by bounded (i.e., limited) rationality (von Neumann and Morgenstern, 2007; Dayan, 2012; Gershman et al., 2015).

## Neuroscience investigating mechanisms has important blind spots

Much neuroscience research can be seen as a site of reproduction of neoliberal capitalist ideology, defining the “optimally” functioning human as a successful market-decision-maker. However, it would be misleading, as well as unethical, to neglect the study of the harmful effects that this perspective has on brain development and on the well-being of real-life individuals. Economic inequality, deprivation and discriminatory exercise of power are almost never seriously considered in *mechanistic* neurobiological studies of mental health, which often take socioeconomic influences as nuisance variables to be controlled for, if considered at all. As an illustrative example, Xiang and co-workers found that control participants displayed deep “theory of mind” when playing with each other a game requiring trust (Xiang et al., 2012). The control participant's “theory of mind” deteriorated when they played with patients with borderline personality disorder, the maladaptive cognition of the latter being the focus of that paper. However, when healthy people *matched for socioeconomic status to the BPD participants* played with the same control subjects, the same deterioration was induced. Yet, instead of concluding that something important was found about the (underprivileged) stratum from which BPD participants came, and pursuing this research, the authors concluded that “*lower SES may be one source of influence for the incapacity of the Borderline subjects to sustain cooperation with their investor partners*”. Instead of examining how lived experience may have given rise to key findings, why the patients with psychological problems may come from these “lower” social strata, and whether there are legitimizing rather than pathologizing ways of understanding the apparent suboptimality of “lower” strata, most neuroscience research unnecessarily sticks to a blinkered evolutionary - medical analytic and interpretative framework.

It is a matter of great importance for public health to research at least two key hypotheses here. First, that placing people in a competitive context distorts their reward and social brain functions toward evaluating actions with no regard to “caring and sharing”, but only “resource control” (Gilbert, 2021). This legitimizes the suffering of their competitors, employees and bosses; and, second, that living under competitive threat and repeatedly experiencing “being a loser” has a detrimental effect on the brain.

As introduced in Table 1, we discern: 1) *deprivation effects*, associated with the marketization of essential needs, at great cost to the well-being of those priced out or continuously at risk of being priced out; 2) *isolation effects*, associated with the behavioral incentive structure, built to favor individualist competition and stigmatizing any “uncompetitive” individual unwilling or unable to conform. 3), intersectional effects that include deprivation and isolation due to belonging to a particular minority or population group; relatedly, 4) Stigma and discrimination are included here, although we acknowledge that they are not exclusively resulting from capitalism and they are particularly exacerbated when intertwined with systematically oppressive structures such as colonialism, white supremacy, patriarchy, hostility to sexual and gender minorities, etc.

### Neurodiversity and neuroplasticity

Consonant to the above, much neuroscience has bolstered medicalization of human suffering and dissemination of singular biological explanations for complex psychosocial problems. Neuroscience thus came to collide (and collude) with systematic and systemic^2^ psychiatric practice, which in theory recognizes the importance of biopsychosocial understanding, but in practice is often reduced to biomedical management (Bentall, 2009). Such management is fueled by the psychiatric drug market, which over-promotes psychiatric diagnoses, incentivizes doctors to overprescribe (Duncan and Marsh, 2021), and has biased their knowledge through academic practices (Matheson, 2016). Two assumptions have resulted, which we challenge in order to reclaim a more balanced and emancipatory neuroscience.

The first is that researching the investigation of the biological underpinnings of behavior discounts rather than informs the rich relational and experiential foundations of mental health. This is challenged by considering neurodiversity.

The second is that neurobiology deterministically creates and solidifies the stigmatizing discourse around the brain, e.g., mental health difficulties arise from brain defects that people cannot recover from without biomedical intervention. This is also challenged by considering neuroplasticity.

#### Neurodiversity

According to Amartya Sen and Martha Nussbaum (Nussbaum and Sen, 1993), human minds afford a diverse spectrum of capacities. Capacities provide for the “beings” and “doings” that people can achieve when given the *real freedom*, namely the necessary means to pursue their goals. However, neuroscience, psychiatry, and psychology are currently dominated by a restrictive discourse of “maladaptation” and “functioning” as an organizing principle to discuss “mental health”, underpinned by the neoliberal-capitalist stigmatization of reliance on public services as unhealthy (Banerjee et al., 2017). The imperative to “function independently”, with limited support from one's community and the welfare state, has infiltrated mental health services, clinical practice and mental health research (Ramon, 2008; Sugarman, 2015, 2019). This pathologizes and stigmatizes those deemed financially dependent, with likely dire consequences for their mental health.

We thus privilege the linguistic and conceptual framework of thinking about psychological wellbeing and its bio-psycho-social correlates that has been pioneered by the neurodiversity movement (Kirmayer et al., 2015; Craine, 2020; Kapp, 2020). On the one hand, a notion of “neurodiversity” has broad appeal, if simply defined as the infinite variety of brain structure and function found among people. On the other hand, the neurodiversity movement asserts that those who diverge from the statistical norm along specific dimensions such as “autism” or “schizotypy” do not need to have their mind cured or made adaptive, but have a legitimate claim to a fulfilling life as they are.

Additionally to the core insights offered by the neurodiversity movement, we caution that legitimizing psychiatric diagnoses through efforts to pinpoint their neural basis is problematic, in view of the robust empirical evidence that the validity of such diagnoses is poor (Insel et al., 2010; Cuthbert and Insel, 2013; Peng et al., 2021). Happily, the current nosological construct of e.g., *schizophrenia* is not a pre-requisite in order to assert divergence along a dimension of schizotypy, or “schizophrenia spectrum” (Hoffman et al., 2007; Villeneuve et al., 2010). Hence we can follow the lead of the neurodiversity movement, to claim that mental well-being should be defined by individuals embedded in their communities, whether or not they identify in terms of diagnosis-based spectra. The basis of current diagnostic criteria is the level of impairment (or “age-appropriate functioning”) in several areas of life (e.g., social, professional, family etc). In practice, this closely links diagnosis to the neoliberal imperative of financial independence. In contrast, in our neurodiversity framework, people who hear voices and are “economically unproductive”, may be perfectly healthy. This claim stands in contrast to mental well-being expected on the basis of orthodox discourses, especially the economically defined rational, self-interested, and efficient market agent. In other words, we reclaim and politicize mental health as “recovery” (Davidson, 2016) or “to love and to work” (McCabe and Daly, 2018). These are aspired to by many mental health professionals, but have been dwarfed by capitalist institutions.

We hypothesize that definitions of mental well-being reclaimed by individuals embedded in their communities can show how the capitalist norm imposed by the everyday discipline of the market harms the neurodiverse and neurodivergent (Morioka et al., 2019; Craine, 2020). A key corollary of stakeholder communities defining what mental health is and the relevant priorities is that researchers (experts by experience in measurement and analysis) should work together with stakeholders (experts by experience of ill health) to operationalize relevant aspects of mental health into the best research assessment procedures (Humm et al., 2020).

#### Neuroplasticity, or the human brain's potential for growth and healing

Within the important constraints dictated by its neurodiversity, the individual brain is plastic, capable of sculpting itself in interaction with its environment. It is an inferential, roughly Bayesian brain, which builds models of the self-in-the-environment, thereby anticipating the consequences of observations and of actions. Such anticipation is closely guided by the engrams of powerful experiences^3^ (Okuyama, 2018). Harmful engrams are inscribed by “toxic” environments - be it family, educational setting, work, a virtual community on social media or general societal factors like gender or race (Ziegler et al., 2020; Zadow et al., 2021). What is also true is that the brain can adjust to an improved, safer, caring environment whether it is due to lower levels of stress or through learning and enhanced problem-solving skills, through a different self-representation etc. (Leventhal and Brooks-Gunn, 2003; Linden, 2006; Osypuk et al., 2012).

This multidirectional process may operate for the better or the worse to shape the self and its brain. Ian Hacking (Hacking et al., 1999), for example, hypothesized that a diagnosis —such as that of depression— may interact directly with the biology related to the condition diagnosed, through the changes of behavior that it can instigate (Slaby, 2010). On the other hand, empirical evidence indicates that psychotherapy, including mindfulness practice, changes the brain (Linden, 2006; Allen et al., 2012; Lane et al., 2015). There is a need to further understand how much of this change takes place in the therapy itself, and how much through bidirectional changes between patients and their environments, that allow them to receive support i.e., through reconstructing the meaning of past experiences and/or through improving their environments by managing stress.

It is important to highlight a caveat regarding neuroplasticity. The philosopher Cathrine Malabou points out that the brain's plasticity can be easily construed as a demand for quick and intense adaptation, e.g. to a work environment (Malabou, 2009). This would result in yet another unattainable standard and conceptual definition of mental health. Along the same lines, the sociologist's Zygmunt Bauman's work on liquid modernity (Bauman, 2000) suggests that increasing globalization, privatization of services and the information revolution have resulted in an environment that demands an individual flexibility that instigates great anxiety and fear^4^. We note that the status anxiety hypothesis, whereby anxiety is fueled by social ranking and social mis-trust (Wilkinson and Pickett, 2006; Burns, 2015), and the social capital hypothesis (Kawachi et al., 1997; Burns, 2015) have not been adequately explored in neuroscience. These provide a helpful framework in order to study not only economic inequality, but also the effect of anxiety and fear due to inequality on brain and behavior.

### What emancipatory neuroscience should be

As neuroimaging research has already linked economic disadvantage with changes in brain structure and function (Hao and Farah, 2020), neuroscientific research must now urgently and rigorously address questions specific to the detailed processes realized by neoliberal capitalism, rather than just deprivation or other fragmentary socio-economic outcomes.

To arrive at this, we advocate a neuroscientific metaphor different from the “rational decision-maker”, namely the human brain as the builder of models of self and others (Chater et al., 2006; Otten et al., 2017). Brains as neurodiverse constructors of generative models seek to model how human relations are generated, “who I need to be and who I can be in my community”. Building on second-person neuroscience (Schilbach et al., 2013; Schilbach, 2016), we claim that this self-and-others approach can direct more ecologically valid and humane research than the selfish rationality approach.

### Specific proposals for further research

In epidemiological mental health and neuroscience, excellent inroads have already started to be made in researching the impact of key features of capitalism on mind-brain health, in that measured “poverty” is rarely poverty in the sense of the World Bank definition, i.e. “not having enough material possessions or income for one's basic needs”; it is relative poverty, such as family income below a certain proportion of the median for a country, or a deprived neighborhood environment (Fry, 2010). They are thus about structural inequality, both a major outcome of and (supposedly) a key motivational engine in capitalism (circular causation). First, therefore, such approaches should be widely expanded and systematically planned, to assess rigorously the impact of the *application of capitalist principles* upon neural development and mental health, especially (1) privatizing welfare state functions such as housing, health and education, (2) austerity measures and cuts in funding (3) IMF, EU, and related public spending policies imposed on countries (4) socio-economic differences between rich and poor countries (5) longitudinal boom-bust cycles.

Second, we propose that human neuroscience must test the set of hypotheses whereby well-known laboratory animal models of stress causing behavioral and neural toxicity may apply in corresponding ways to the stressors that many people encounter in free-market societies (Table 2). This would have important implications for policy too. Many species rely on complex networks of social interactions which have been interpreted as regulated by a dominance psychobiological system (Johnson et al., 2012), to compete for social roles and resources, although this is not a “law of nature” (Mech, 1999). Important questions arise for neuroscience: first, can the relevance of the dominance system and its interaction with varying socio-economic factors (e.g., deprivations, isolations etc.) be rigorously tested in humans, and can the effects of these interactions on brain health be measured? If this is so, policy initiatives could go beyond balance of power (establishing rights), activating instead the affiliative systems rewarding helping relationships, and implementing *inter-personalized mental health* policies (Gilbert, 2021; Bolis et al., 2022).

**Table 2:** Animal paradigms and their hypothesized human analogs.

**Animal model**	**Psychobiological human research**
Chronic mild stress	Chronic socioeconomic disadvantage
Social defeat	Market life events (loss of occupational identity, employment, housing)
Variable foraging demand	Precarity
Activation of “resource control” vs. “caring and sharing” interactions	Effects of imposing market competition on the balance of valuing own vs. others' needs.

Third, we propose that all human neuroscience purporting to investigate mechanisms important for mental wellbeing must be powered (both in the statistical and social sense) to relate mechanisms to real, contextualized lives. Rather than ignoring or controlling for socio-economic conditions, gender, intersectionality etc., research should collect high quality data and be designed to answer whether apparently maladaptive factors have resulted from lived experience within market economies. Such contextualized and, ideally, longitudinal data should include the voice of those lacking privilege, both in co-designing and interpreting the relevant components of research. Mental health and neuroscience research should be designed to influence societal change, rather than focus on individual maladaptation.

Fourth, we propose that mental health and neuroscience research should ask how the mind-brain deals with, and is affected by, systems and relations of power as exerted by those who control financial and social resources, upon those who have much less. In neighboring fields outside neuroscience, there are some examples of that type of work. At the psychological level, the Power-Threat-Meaning-Framework (PTM) formulates both negative and positive notions of power, and proposes an alternative to individualistic diagnoses. It aims to conceptualize the role of power in forming personal meaning and facing psychological distress (Johnstone and Boyle, 2018). Prior to PTM, other psychotherapeutic approaches have aimed to specifically address the issue of social justice. These include feminist counseling (Ross, 2010), and services aiming to create gender and cultural accountability such as “Just Therapy” clinic in Australia (Tamasese and Waldegrave, 1994). Neuroscience could contribute to these emancipatory psychologies, to develop an integrated understanding about how neurodiverse structure and function mediates the relationship between living conditions, personal experience and behavior. It should also aim to test the claims of important contributions such as the PTM.

## Concluding remarks: researching a post-capitalist mental health

Empirical testing of hypotheses regarding the risks that capitalism poses to the mind and brain is paramount, but there is also a need for general principles, for how to conduct neuroscience that goes beyond assuming the norms of capitalist societies. We thus propose moving away from the capitalist framing of pathology based on maladaptive decision-making within a taken-for-granted, good-enough, normal market society. We propose a post-capitalist framing of pathology which highlights failures of society to provide good-enough communities, wherein individuals can both fashion and fulfill their needs. This proposed shift in the emphasis on health and pathology for the purposes of research is illustrated in Table 3.

**Table 3:** Proposed shift of research framing from (maladaptive) market agent to (ill-provided-for) neurodiverse community member.

**From the individualist, market-oriented**	**To a community-oriented**
Researching new individualistic diagnoses	Scientifically based formulation/conceptualization of mental distress as the effect of a harmful context on a neurodiverse individual
Well-adjusted to norms	Expressing neurodiversity
“Healthy behavior” derived from Western, rich, young, highly educated research participants	Healthy behavior as affordance to fulfill need in an appropriate niche when given the appropriate support/assistance
Assumption of independence	Interdependence
Therapy for symptom alleviation (People adapt to their environment)	Therapy to empower for justice (People change their environment incl. through alleviating symptoms)
Most efficient remission in terms of positivist mental health measurements	Collaboratively seeking improvement through treatments
Biological interventions marketed as cure (vs reality of symptom control or debilitating effects)	Collaborative biological interventions in the service of recipient empowerment to achieve their own goals, as opposed to the current so-called recovery model which distorts patient's true goals into abilities to adapt to the requirements of the market.

Finally, we propose to maximize the voice of the under-privileged in the design and conduct of all research purporting to subserve mental health. This would shift the dynamic of research from the replication of the hierarchy of “the expert that performs research onto the participant” to a more collaborative and horizontal relationship. There is considerable work being done on this, but a further shift of researcher attitudes and research funding structures is critical (Pincham et al., 2020).

Here, we draw on the tradition of Participatory Action Research (PAR) and the work of Paulo Freire in critical pedagogy (Baum et al., 2006). This involves reflection upon the nature of knowledge and the extent to which knowledge can represent the interests of the powerful and serve to reinforce their positions in society. It affirms that experience can be a basis of knowing and that experiential learning can lead to a legitimate form of knowledge that influences practice. In the same vein, following the more recent trend of including Experts By Experience in service improvement, we suggest that psychology and neuroscience research projects should if at all possible be co-designed with individuals with lived experience (Baum et al., 2006; Humm et al., 2020), survivors of harm (including as caused by services), service users, front-line workers and other key stake-holders.

## Author contributions

DK, AH, BP, and MM conceived of the presented idea. DK, IS, VF, AH, BP, LD, and MM developed the theoretical framework. DK, IS, AH, VF, and MM performed the literature reviews. WE-D, LD, and MM provided guidance and supervision. All authors discussed the results and contributed to the final manuscript.

## Footnotes

^1^“Optimal” in the sense of “the best practicably possible”, not in the sense of “perfect”.

^2^Systematic because it is widespread and defined by policies, guidelines and standard procedures and systemic because it doesn't necessarily reflect individual psychiatrists'/mental health workers' views.

^3^Engrams may result from recent or developmental personal experiences, but also from historical experience engraved in genes or cultural memes. This provides a lens through which to consider neurodiversity as sets of expectations both about both “how things are” and “what is needed”.

^4^Bauman is very far from being a neuroscientist, yet in neuroscience anxiety and fear behaviors are recognized as the normal responses to potential and present threat, respectively. Translating to contemporary capitalist context, it would be expected for people facing frequent economic threats to live in anxiety and fear.
