# Supplementary material for: Testing hypotheses about the harm that capitalism causes to the mind and brain: a theoretical framework for neuroscience research
Source: Front Sociol. 2023 Jun 19;8:1030115. doi: 10.3389/fsoc.2023.1030115 (PMC10315660; doi:10.3389/fsoc.2023.1030115)
Supplement: Supplementary file 1 [file Table_1.DOCX]

**Supplement to**

**Testing hypotheses about the harm that capitalism causes to the mind and brain: a theoretical framework for neuroscience research**

Danae S. Kokorikou, Ioannis Sarigiannidis, Vincenzo G. Fiore, Beth Parkin, Alexandra Hopkins, Wael El-Deredy, Laura Dilley^^[[1]](#footnote-1)^^ & Michael Moutoussis^1*^

# **Critical Glossary**

The definitions that we use are rooted in the lived experience of the authors, as mental health service users and providers. This glossary tells the reader what we mean, but also warns that when one uses terms such as ‘patient’, ‘madness’, ‘health’, etc., one refers to ambiguous, debated, historically loaded terms that have different meanings for different stakeholders. To take an important example^^[[2]](#footnote-2)^^, many people seeking psychotherapy are happier to be called ‘patient’ than ‘client’, others the opposite, depending on the meaning they attach to these terms. We give an idea of what the terms refer to, but we underline that there is no consensus about the meaning, nor the values, that these terms refer to, and we admit a clear defeat in the endeavor to arrive at definitions accepted by key social groups invested in using these terms.

| **Term** | **Definition** | **Historical usage, caveats and controversies** |
| --- | --- | --- |
| Capitalism | Legal protection of the freedom to own property, and to buying and selling labor, goods and services for the purposes of making monetary profit. | Capitalism privileges the *freedom to* exert financial power, pursue private profit and accumulate capital, over the *freedom from* need. |
| Disease | Biological process damaging the healthy function of part or parts of the body | Term often used to justify researching and treating madness by biological means, based on the early success of preventing and treating cerebral syphilis, and of researching and treating other states based on analogous hypotheses about the brain. It is a peripheral term to the dominant biopsychosocial model of psychiatry. |
| Health | State of structure and function of a part or parts of the body that sustains its biological function. | Whereas this definition has a simple interpretation for externally assessed organs, e.g., a ‘healthy’ heart can sustainably maintain blood flow that adequately perfuses the body, porting the term to ‘mental health’ is much more problematic. |
| Illness | State incompatible with health of the whole person. |  |
| Madness | Extreme deviation from neurotypical perception, rationality and action, to a degree that ordinary individuals without medical or psychological training would recognize as highly maladaptive. |  |
| Mental Health |  |  |
| Mental illness | Literally, subjective state incompatible with mental health in an individual who did not previously display this state. In practice, state of fulfilling criteria for a mental health disorder. | Crucially, not a term used by classifications of mental disorders. For example, the ICD-10 made specific reference to *not* using it. However, it is used widely with the implication that psychiatrists are doctors like all others, the conditions they treat are bodily problems like, say, diabetes, etc. |
| Neoliberalism | Contemporary form of capitalism with a strong state promoting financialization and private enterprise, while striving to move all profitable economic activity to the private sector. | The term is mostly defined and used by the critics rather than the subjects of the free-market beliefs in question. |
| Severe Mental Illness | Term denoting that specialist psychiatric services are responsible to manage high risk and/or highly divergent experience and behavior. Closely associated with psychosis. | Term is used to ration services (so that most people who don’t have ‘SMI’ have no access to highly trained staff) but also to reinforce highly questionable categories such as ‘schizophrenia’ and the biomedical model of their management. |
| Poverty | World Bank definition = Not having enough material possessions or income for one’s basic needs. |  |
| Psychiatry | The branch of medicine centrally concerned with treating the mad, but which also applies the tools developed in the process to any form of psychological suffering that may benefit from said tools. The large bulk of psychiatrists do not adhere to a medical but to a biopsychosocial model of the problems they deal with. | This is another term which is in practice circularly defined - psychiatry is what psychiatrists are given to do by society, which does not use a singular criterion but a network of interests to allocate, or not, authority to psychiatrists. Crucially, organizations such as the Royal College of Psychiatrists do not define the term. |
| Patient | The recipient of (mental) health services. | The term ‘patient’ has been associated with passive subjugation to paternalistic interventions. A number of alternatives have been used, such as ‘client’, ‘service-user’, ‘help-seeker’, ‘clinical subject’. None is satisfactory. |

**Table S1** Critical glossary

1. These co-authors contributed equally to the paper [↑](#footnote-ref-1)
2. We thank the anonymous reviewer for this example. [↑](#footnote-ref-2)
